# A cyclic swarming assay produces genetically and phenotypically distinctive variants of *Enterobacter* sp. SM3

**DOI:** 10.3389/fmicb.2026.1968333

**Published:** 2026-10-28

**Authors:** Dennis D. H. Kim, Francesco S. Pagano, Stephen K. Costa, Danielle A. Germann, Peter Belenky, Jay X. Tang

**Affiliations:** 1 Department of Physics, Brown University, Providence, RI, United States; 2 Department of Molecular Microbiology and Immunology, Brown University, Providence, RI, United States

**Keywords:** bacterial swimming, biofilms, chemotaxis, *Enterobacter sp*. SM3, gut microbiome, hyper-flagellation, hyper-swarming, swarming motility

## Abstract

**Introduction:**

Swarming motility, the collective migration of bacteria across semi-solid surfaces, is often associated with cell elongation and increased flagella number or strength. *Enterobacter* sp. SM3 is a strain originally isolated from murine gut and previously shown to mitigate colitis in mice.

**Methods:**

To investigate phenotypic and genomic correlates of enhanced surface motility, we derived hyper-swarming variants of SM3 through serial passage from a single clonal isolate, generating five independent lineages collectively termed “Swarmium.”

**Results:**

Optical microscopy revealed that Swarmium populations contained cells that were two to three times longer than the parental strain. Motility analysis showed similar swimming speed and tumble duration, but longer run times and reduced tumble angles. Whole-genome sequencing (mean coverage 634×; range 571–845×) identified population-level sequence variants affecting loci annotated for functions including flagellar regulation (rcsB), cell division (ftsL), translation machinery (ribosomal RNA operons and EF-Tu), and Type VI secretion-associated loci (including PAAR-adjacent regions and DUF6531-containing proteins).

**Discussion:**

These findings identify candidate genes associated with the hyper-swarming phenotype, although their functional significance remains unclear. This study identifies genetic changes in populations selected based on swarm expansion rate and provides useful leads for future functional and mechanistic investigations of bacterial swarm motility.

## Introduction

Bacterial swarming motility is a collective form of surface migration driven by flagella and coordinated multicellular behavior (Turner et al., 2010; Kearns, 2010; Partridge and Harshey, 2013; Ariel et al., 2015). In contrast, bacterial swimming motility refers to the movement of an individual bacterium in liquid, propelled by its own flagella (Berg and Anderson, 1973; Grognot and Taute, 2021). For peritrichous bacteria, the swimming motion manifests run-and-tumble dynamics, in which straight “runs” are intermittently interrupted by reorientation events (“tumbles”) mediated by reversal in rotation direction of one or more flagella (Berg, 2003; Johnson et al., 2024). Swarming involves additional layers of regulation, including cell-cell interactions (Partridge and Harshey, 2013; Darnton et al., 2010), surface sensing (Partridge and Harshey, 2013; Patrick and Kearns, 2012; Lee and Belas, 2015; Pawul et al., 2024), increased secretion of osmolytes or surfactants (Ping et al., 2014; Yang et al., 2017; Bru et al., 2023) and physiological adaptations such as cell elongation (Kearns, 2010; Allison et al., 1992b; Copeland and Weibel, 2009), increased number of flagella (Kearns, 2010; Partridge and Harshey, 2013; Allison et al., 1992b; Kearns and Losick, 2003), and reduced frequency of tumbles (Johnson et al., 2024; Partridge et al., 2019, 2020).

Swarming has frequently been associated with pathogenicity (Allison et al., 1992a; Mobley and Belas, 1995), biofilm formation (Verstraeten et al., 2008), and antibiotic resistance (Overhage et al., 2008). The functional role of swarming is varied, and the behavior is found in both pathogenic and non-pathogenic bacteria (Partridge and Harshey, 2013; Sharma and Anand, 2002). Notably, *Enterobacter* sp. SM3, a strain isolated from the murine intestinal lumen, has been shown to reduce inflammation in mouse models of colitis (De et al., 2021), demonstrating that swarming can also be linked to beneficial host interactions rather than virulence. In well-studied model organisms such as *Bacillus subtilis*, the regulatory pathways governing swarming—encompassing flagellar biosynthesis, surfactant production, and cell differentiation—have been extensively characterized (Kearns, 2010; Partridge and Harshey, 2013). In contrast, the genetic and regulatory basis of swarming in *Enterobacter* species remains largely undefined. In particular, for *Enterobacter* sp. SM3, there has not been much study on how genetic variation influences motility behavior, morphology, and collective surface migration. Furthermore, although previous studies have shown that swarming populations often exhibit altered motility parameters, including reduced tumble frequency and smaller reorientation angles, which may lead to more persistent trajectories (Ariel et al., 2015; Johnson et al., 2024; Partridge et al., 2020), whether these behavioral changes arise from specific genetic determinants or represent emergent properties of physiological adaptation remains unclear.

In this study, we generated elongated populations of *Enterobacter* sp. SM3 through serial passage on semi-solid agar containing growth medium and characterized their morphology, motility, and genomic variation. Our initial approach did not isolate clonal mutants but rather examined genetically heterogeneous populations generated through repeated selection at expanding swarm fronts. We identified several population-level genetic variants exhibiting observed phenotypic changes and identified these variants as candidates for future investigation of phenotypes caused by mutations at specific loci in the genome of this organism.

## Materials and methods

### Isolation of a clonal parental strain (SM3A) by single-colony purification

*Enterobacter sp*. SM3 was originally isolated from the murine intestinal lumen as described previously (De et al., 2021). Frozen stocks of SM3 were obtained from the laboratory of Dr. Sridhar Mani at the Albert Einstein College of Medicine (Bronx, NY). An overnight culture of SM3 was grown in lysogeny broth (LB) at 32 °C. The culture was diluted 10^7^-fold in a simple motility buffer (67 mM NaCl, 0.1 mM EDTA, and 10 mM sodium phosphate at pH 7.0). Then, 300 μL of the dilution was spread evenly onto 1% (wt/vol) agar LB plates to achieve well-separated colonies. Plates were incubated for 16 h at 32 °C and 50% relative humidity. A single, well-isolated colony was randomly picked and designated SM3A, the clonal parental strain used for subsequent experiments.

### Serial passage to generate hyper-swarming variants

A fresh culture of SM3A was prepared by inoculating LB and incubating for ~4 h to an optical density at 600 nm (*OD*_600_) of ~1.50. Unless otherwise stated, the growth condition in the liquid medium was set at 32 °C and 160 rpm aeration.

Serial passage experiments follow a procedure reported previously (Zhang et al., 2022), as illustrated schematically in Figure 1. In a typical experiment, 7 μL of the fresh growth was inoculated at the center of a 0.5% (wt/vol) agar LB plate. The plate was incubated for ~6 h to achieve active swarm-front expansion, to the extent where around two-thirds of the plate was covered with the growing colony. Using a sterile pipette tip or inoculating loop, a small patch was collected from the swarm front and transferred to the center on a plate pulled with 10 ml fresh 0.5% LB agar. This transfer with the subsequent bacterial growth and expansion on the plate, denoted hereafter as a “passage,” was repeated 5 to 6 times. After the final passage, material from the swarm front was picked using a pipette tip and inoculated in LB for fresh growth before storage in a −80 °C freezer for preservation of the variant generated. In the meantime, microscopy experiments, as detailed below, were performed with the material directly picked from the swarm front of the final passage. No single-colony purification was performed following serial passage; therefore, each resulting population may contain multiple genotypes that arose during the passage. Five independent variants, collectively referred to as SM3 Swarmium (SM3S), were generated and analyzed by this procedure. They were designated as SM3SA, SM3SB, SM3SC, SM3SD, and SM3SE, following the chronological order of the experiments performed.

**Figure 1:**
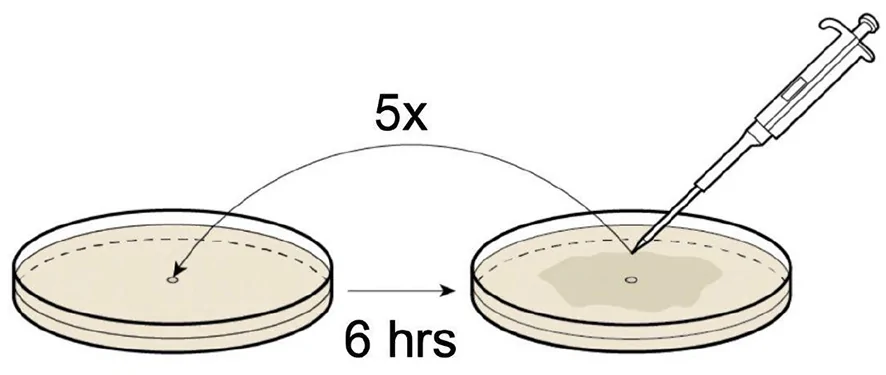
A simplified illustration of the serial passage procedure. A small patch of bacteria at the swarm front is picked by a sterile pipet tip **(Right)** to inoculate another plate at its center **(Left)** to start a new cycle of growth and expansion. The initial inoculation was done with a droplet of fresh liquid culture placed at the plate center, which is not shown in the illustration for simplicity.

### Microscopy and cell length measurements

Bacteria from the swarm edge after serial passages were suspended in the motility buffer and imaged by light microscopy for cell-length measurements. Specifically, roughly 5 μL of material was picked using a pipette tip from the swarm edge and diluted into 100 μL of the same motility buffer as described above. After gentle vertexing, a 7 μL aliquot was taken from the dilution and deposited on a microscope cover slip with strips of vacuum grease applied on the edges. A microscope slide was then mounted on the top, pressing the droplet into a thin layer of liquid so that most bacteria end up sticking to the glass surfaces. The sample was viewed under an inverted microscope, Nikon TE-2000, using a 40 × magnification objective lens. Images were captured using a digital camera (Point Gray GS3-U3 Grasshopper3). Measurements of cell length were performed using the “measure” feature in ImageJ.

### Motility analysis by a custom Python algorithm

The same microscopy procedure as described above was performed, with the exception that the coverslip was pressed less against the slide so that most motile bacteria swim freely within a layer of fluid tens of micrometers thick. Short movies were taken at 25 frames per second (fps) or once every 0.04 s for motility analysis. An in-house Python program was used to analyze the motility of SM3A and SM3S variants. A comprehensive kinematic analysis of the bacterial movement was implemented to determine tumble events. Below is a brief recap of the algorithm with slight modifications from what was detailed in a previous publication (Johnson et al., 2024).

First, the algorithm recognizes a decrease in linear speed, marking events where:


$$\begin{array}{l} \frac{\Delta v}{v_{\min}}>\;0.7,\text{where}\;v=\text{velocity}. \end{array}$$

Second, the algorithm detects an increase in angular speed, the rotation of the cell body, that exceeds 20% of the baseline established in the previous frames. Only if both criteria are met is the event identified as a tumble event.

The duration of a tumble is calculated by counting all the neighboring frames, which satisfy the inequality:


$$\begin{array}{l} v\left(t\right)\leq \;v\left(t_{min}\right)\;+\;0.3\Delta v,\text{where}v\text{is velocity and}\;t\;\text{is time}. \end{array}$$

This dual-threshold method minimizes false positives and ensures that the recorded tumble events actually reflect the bacterial behavior, but it may miss very brief tumble moments if they occur faster than the frame interval of 0.04 s. This algorithm produced linear velocity, run time, tumble angle, and tumble duration to characterize the motility of SM3A and its SM3S variants.

### Whole-genome sequencing, assembly, and variant analysis

Genomic DNA from SM3A and its Swarmium variants was subjected to Illumina short-read sequencing (*de novo* workflow; library construction and sequencing performed by Novogene). Raw reads were assembled *de novo*, and assemblies were curated using the Bacterial and Viral Bioinformatics Resource Center (BV-BRC). Assembly statistics (assembly length, contig count, GC content, coding ratio, predicted CDS, tRNA/rRNA counts, and N50) were recorded for quality assessment. Illumina paired-end (2 × 150 bp) sequencing on a NovaSeq 6000 platform produced between 18 and 27 million read pairs per genome, yielding 571–845 × coverage per genome (Table 1).

**Table 1:** Genome assembly coverage for *Enterobacter sp*. SM3 and its Swarmium variants.

Variant	Genome assembly coverage (×)
SM3A	616
SM3SA	586
SM3SB	577
SM3SC	571
SM3SD	845
SM3SE	608

Average sequencing coverage values (×) were calculated as total clean bases generated by Illumina paired-end (150 bp) sequencing divided by the total assembled genome length (~4.8 Mb per strain). Coverage values range from 571 × (SM3SC) to 845 × (SM3SD), reflecting read yield differences among sequencing runs but all exceeding 500 ×, which ensure high-confidence base-calling and variant detection.

### Annotating assemblies and identifying polymorphisms

To characterize genomic variation arising in populations subjected to repeated swarming selection, assemblies were annotated with Bakta (version 1.10.3) using a full database (version 5.1). Single-nucleotide polymorphisms (SNPs) and small insertions/deletions (indels) were identified using MUMmer 3 (version 3.23). Briefly, Swarmium population assemblies were aligned against the wild-type *Enterobacter* sp. SM3A assembly using the “nucmer” utility. Redundant and overlapping alignments were quality filtered for one-to-one matches and polymorphisms, then extracted from filtered alignments. Extracted polymorphism sequences were then referenced against the annotated assemblies to identify the location of polymorphisms in the *Enterobacter* sp. chromosome. This identified and annotated SNPS and indels in the affected loci. Putative functional impacts were inferred from gene annotations (including motility regulation, cell division, translation, and interbacterial competition systems). Where applicable, protein-level consequences of coding variants were explored by predictive structural/functional assessment of the mutated sequences.

## Results

This study began from a clonal isolate of SM3, named SM3A, which was randomly picked from a single colony on a hard agar plate. The ensuing experiments were designed to test the hypothesis that SM3 has the propensity to produce variants with enhanced-swarming phenotypes based on findings of a preceding study on SM1, a similar strain of *Enterobacter* (Zhang et al., 2022). \Serial passages on 0.5% LB agar yielded five independent variants from the single clonal parent (SM3A). These variants were conveniently labeled as SM3 Swarmium A-E (SM3SA-E). The single clone origin of SM3A provided a defined ancestral background from which five independent populations were generated. However, because the resulting populations were not single-colony purified following the five serial passage experiments, they represent independently selected populations rather than defined clonal mutant strains.

Immediately after the final passage, cells taken from the swarm front exhibit pronounced filamentation, imaged by phase-contrast microscopy (Figure 2A). This property is consistent with a swarming-associated morphology. Cell length distributions further reveal a marked shift toward longer cell lengths in all variants relative to the parental strain, although the extent and variability of elongation differ among the five variants (Figure 2B).

**Figure 2:**
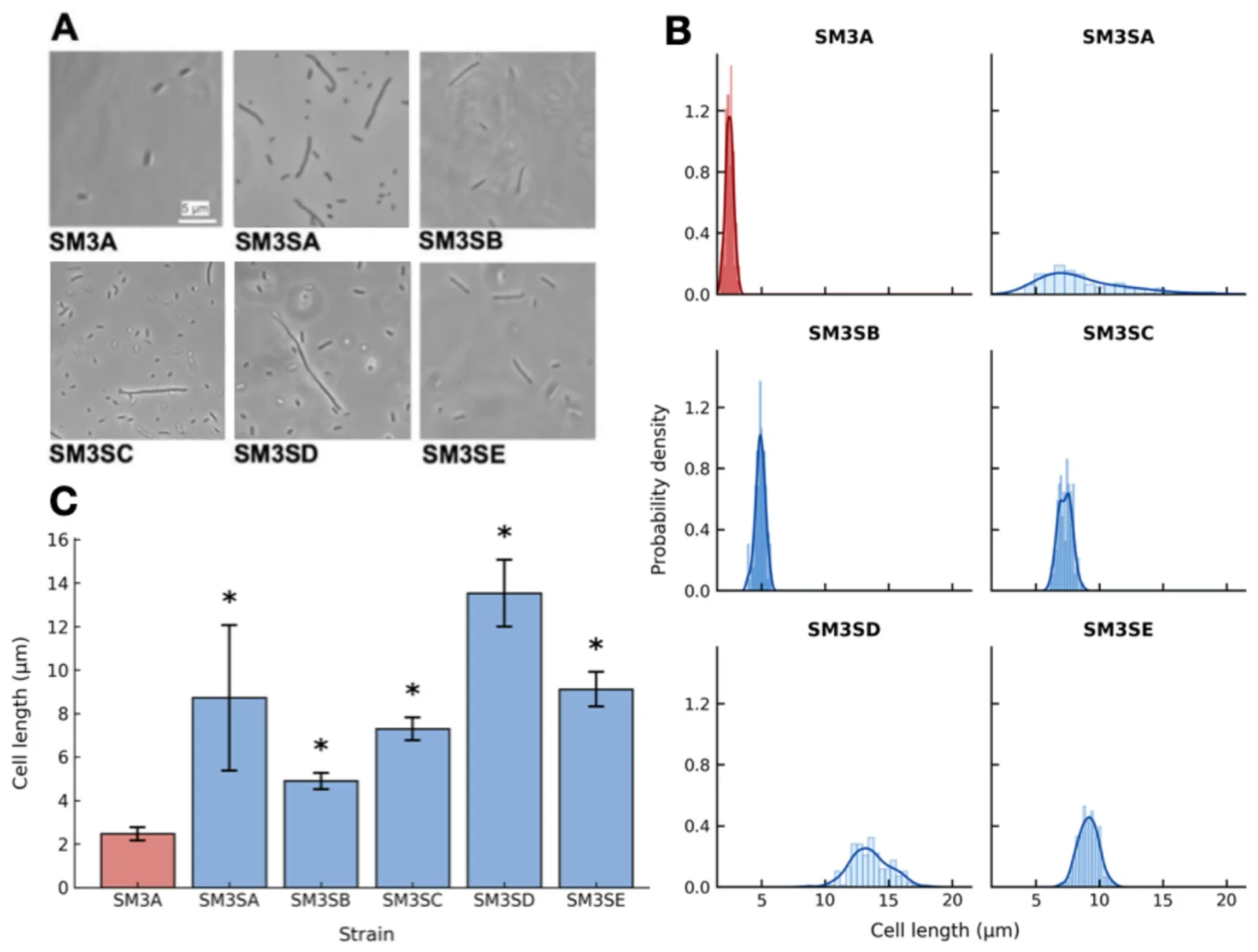
Morphology and cell length characterization of SM3A and its variants. **(A)** Representative phase-contrast images of the parental strain SM3A, and the five Swarmium variants (SM3SA–E) acquired using a 40x objective. A scale bar of 5 μm applies to all images. **(B)** Cell length distributions for each strain. Histograms represent the probability density of measured cell lengths, and solid lines show kernel density estimation fits. **(C)** Comparison of average cell length of SM3A and the Swarmium variants. Bars represent the mean cell length measured from 150–200 cells per strain, and error bars indicate ±1 standard deviation. Asterisks denote statistically significant differences relative to SM3A (*p* < 0.05, one-way ANOVA with *post hoc* multiple-comparison test).

Quantitative measurements (150–200 cells per condition) showed a >3 times increase in mean cell length across the Swarmium variants (7.56 μm) relative to SM3A (SM3A, 2.41 μm). As shown in Figure 2C, one-way ANOVA with *post hoc* testing indicates that all Swarmium variants differ significantly from SM3A (*p* < 0.05) in average length. This elongation result is consistent with a known swarming phenotype, i.e., its propensity to elongate as a selectable trait.

Motility movies (25 frames per second, Δ*t* = 0.04 s) were analyzed with a custom Python tracker to extract instantaneous linear speed, angular speed, run duration, tumble duration, and tumble angle. As shown in Figure 3, the swimming velocity and tumble duration are not significantly different across groups. However, Swarmium cells display longer runs and smaller tumble angles than SM3A, indicating enhanced directional persistence. Breakdown of the comparison among SM3SA–E shows some variation among the Swarmium variants. Although the five Swarmium populations exhibited a common overall phenotype characterized by increased cell length (Figure 2C) and more persistent run-and-tumble behavior relative to SM3A (Figure 3), the magnitude of these phenotypic changes varied among the individual populations. This variation is not surprising, since these variants were isolated from independent trials, each involving multiple cycles of growth and selected for populations of increased swarm expansion.

**Figure 3:**
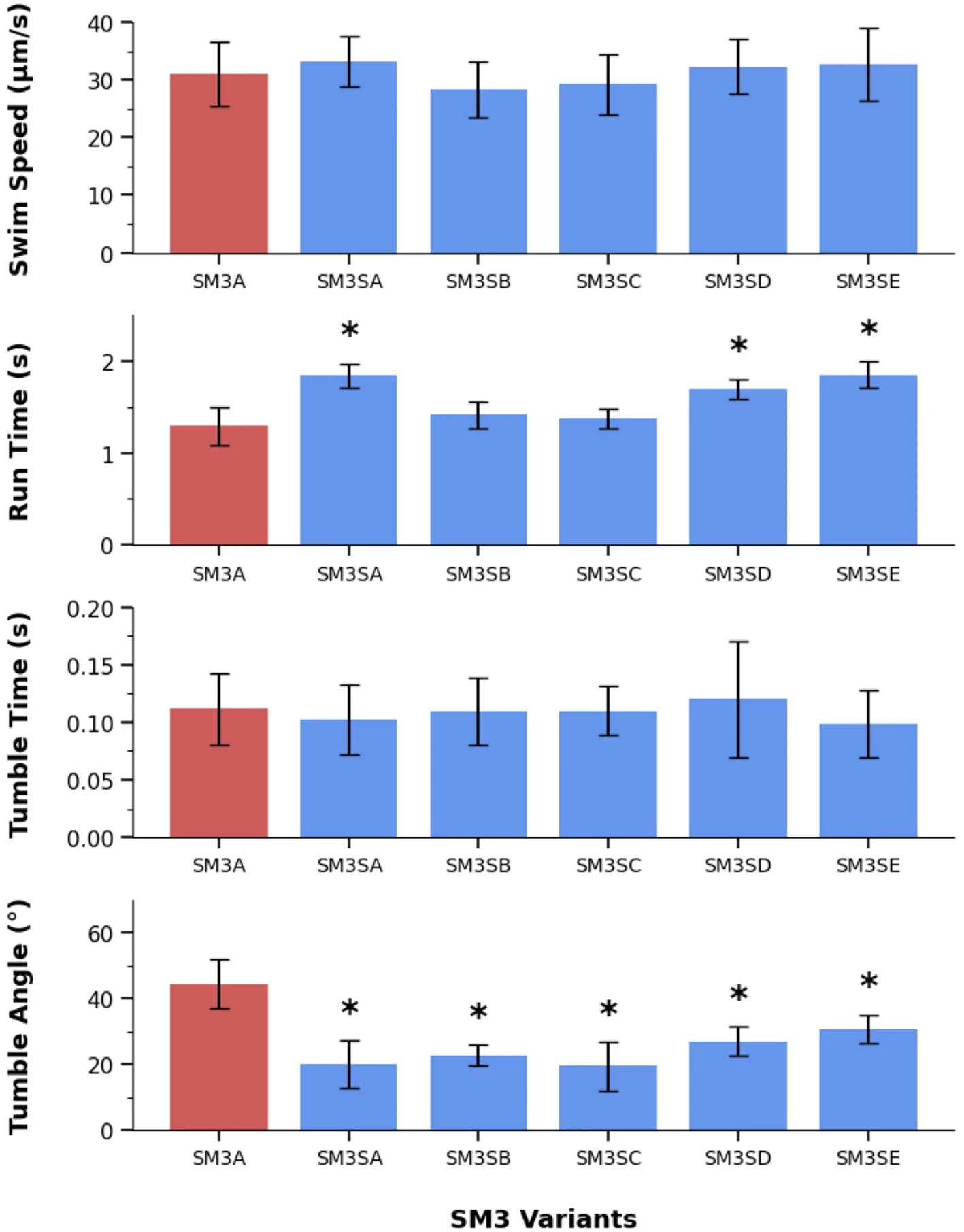
Comparison of kinematic parameters of swimming motility among cells from SM3A and SM3SA-E at the swarm front and diluted with motility buffer. The error bars indicate one standard deviation averaged over 150 data points. The chart shows a comparison of each Swarmium variant on the following measurements of motility: average cell velocity in μm/s; average run time in seconds; average tumble time in seconds; average tumble angle in degrees (°). The values were determined from the microscopy images processed using an in-house Python algorithm. The * indicates a significant difference compared with the average value of the SM3, set with *p*-value < 0.05.

Genetic analysis following complete sequence annotation of SM3A and SM3SA–E provides insights, as well as limitations of the designed study. Variant analysis identified SNPs and indels in genes associated with flagellar regulation (rcsB), cell division (ftsL), translation (rRNA operons and EF-Tu), and Type VI secretion-associated loci. However, coding variants were not systematically classified as synonymous or non-synonymous, and their predicted structural or functional consequences were not determined. Accordingly, we cannot interpret these mutations as causative of the observed phenotypes. Rather, the identified variants represent population-level genomic changes associated with populations selected for increased swarm expansion. These findings highlight candidate loci with potential relevance to the observed changes in bacterial morphology and motility and provide a basis for future functional investigation.

*De novo* assemblies across SM3A and Swarmium variants displayed comparable lengths and GC contents (~4.8 Mb, 56%), though mutants B and E showed slightly lower N50 values and reduced rRNA/tRNA counts (Table 2).

**Table 2:** Summary of DNA sequence data for SM3A and five SM3 Swarmium variants (SM3SA–E).

Genome	Assembly length (bp)	# Contigs	GC content (%)	Coding ratio	#CDS	tRNA	rRNA	N50 (bp)
*Enterobacter sp*. SM3A	4,817,884	57	56	0.897	4,397	78	4	525,398
*Enterobacter sp*. SM3SA	4,816,760	56	56	0.897	4,394	74	4	525,398
*Enterobacter sp*. SM3SB	4,809,068	64	56	0.898	4,390	48	2	320,875
*Enterobacter sp*. SM3SC	4,817,355	56	56	0.897	4,396	75	4	525,398
*Enterobacter sp*. SM3SD	4,817,772	57	56	0.897	4,397	78	4	525,398
*Enterobacter sp*. SM3SE	4,810,167	57	56	0.898	4,390	57	2	357,373

The genomic metrics listed include total assembly length, number of contigs, GC content, coding ratio, number of predicted coding sequences (CDS), number of tRNA and rRNA genes, as well as the N50 value, which serves as a measure of assembly quality.

Variant discovery relative to SM3A revealed different population-level SNP/indel profiles among SM3SA–E (Table 3). SNPs occurred within both coding and intergenic regions, although coding variants were not systematically classified as synonymous or non-synonymous and their functional consequences remain unvalidated. SM3SA and SM3SB exhibited higher SNP counts, whereas SM3SE showed the most indels. The number of genes affected by sequence variation also differed among the Swarmium populations (Table 3), indicating varying degrees of genomic divergence.

**Table 3:** Summary of single-nucleotide polymorphisms (SNPs) and insertions/deletions (indels) identified in each Swarmium variant relative to the parental SM3A strain.

Variant	SNPs	Indels	Genes affected
SM3SA	44	8	9
SM3SB	51	8	7
SM3SC	17	2	6
SM3SD	27	4	7
SM3SE	35	12	11

Examination of the loci containing these variants identified changes mapping to genes annotated for functions involving flagellar/motility regulation (rcsB), cell division (ftsL), translation (rRNA operons; EF-Tu), and interbacterial competition modules (DUF6531 and PAAR-associated Type VI effectors) (Table 4). These functional categories are relevant in the context of the observed phenotypes, including cell elongation and altered run-and-tumble control. Among the populations carrying rcsB variants (SM3SA, SM3SC, and SM3SD), SM3SA and SM3SD exhibited significantly longer run times relative to SM3A. SM3SD also exhibited the greatest mean cell length among the Swarmium populations, while the ftsL variant was unique to SM3SE (Table 4 and Figures 2C, 3). Variants were also identified in regions associated with Type VI secretion, including PAAR-associated loci and regions containing DUF6531. Whereas, DUF6531 is a domain of unknown function, its genomic context adjacent to Type VI secretion modules suggests a possible—but unconfirmed—link to cooperative or competitive surface interactions. Together, these findings identify genomic differences among the independently selected Swarmium populations and highlight candidate loci for future investigation of the genetic basis of the observed morphological and motility phenotypes. Because these variants were not functionally validated, their specific contributions to the observed phenotypes remain to be established.

**Table 4:** A summary of mutated genes identified in *Enterobacter* sp. SM3 Swarmium.

Gene ID	Gene name	Variants affected	Intergenic?
GJPEAO_04534	*rrl*	A, B, D, E	No
GJPEAO_04546	*rrs*	All	No
–	*rrs*	D	No
–	EF-Tu	A, B, C, E	No
–	DUF6531; Rhs	A, B, E	No
–	DUF6531; Rhs	C, D	No
GJPEAO_04544	DUF6531	A, D	No
–	DUF6531	B, E	No
–	*rcsB*	A, C, D	No
–	PAAR	All	No
–	PAAR	B	No
GJPEAO_04120	*fieF*	E	No
GJPEAO_04353	*rbsK*	E	No
GJPEAO_03833	*ftsL*	E	No
GJPEAO_04533	*rrf*	A, B, D, E	Yes
–	–	A, C, E	Yes
–	–	A	No
GJPEAO_04538	–	B, C, D, E	No

The table lists genes found to be mutated across SM3 variants (A–E), including gene identifiers, gene names (with those in regular font if they currently refer to proteins or protein domains/families rather than specific gene symbols), the specific variants in which each mutation was observed, and whether the variation is intergenic (i.e., occurring in non-coding regulatory regions).

## Discussion

This study shows that repeated swarming selection on soft agar produces *Enterobacter sp*. SM3 variants with pronounced morphological and behavioral shifts, consistent with what has been recognized in the microbiology literature on bacterial swarming (Kearns, 2010; Ariel et al., 2015; Partridge et al., 2020). The variants, collectively referred to as Swarmium, display pronounced cell elongation and increased directional persistence, indicating enhanced surface motility. Notably, the elongated morphology, reduced tumble angles (with unchanged tumble duration), and extended run times of the Swarmium variants produce more persistent trajectories without having to vary in swimming speed. Hydrodynamically, longer cells experience greater rotational drag during reorientation; coupled with peritrichous hyper-flagellation, this favors small-angle reorientations without decreasing velocity—an efficient regime for coordinated surface migration (Johnson et al., 2024; Kamdar et al., 2023). The results of this study, combined with the related preceding reports on SM3 (Johnson et al., 2024) and a hyper-elongated mutant of SM1 (Zhang et al., 2022), collectively demonstrate a strong linkage between cell morphology and motility dynamics in populations of *Enterobacter sp*. SM3 selected for enhanced swarm expansion. Given the host-protective role previously reported for SM3 (De et al., 2021), enhanced motility may provide a physiological advantage by facilitating interactions with the intestinal environment.

The population-level sequence variants identified in this report occur in genetic modules with functions relevant to the observed phenotype and therefore highlight avenues for future investigation. De-repression of flagellar genes via *rcsB* perturbation can enhance flagellar abundance and bundle stability during runs; division defects from *ftsL* lesions explain filamentation (Majdalani and Gottesman, 2005; Buddelmeijer and Beckwith, 2004); changes in translation capacity/efficiency (rRNA operon copy number and EF-Tu) can retune proteome allocation toward motility structures (Klappenbach et al., 2000, 2001; Harvey et al., 2019; Ayan et al., 2020); and Type VI-linked effectors (*DUF6531/PAAR*) can bolster competitive fitness at dense swarm fronts (Shneider et al., 2013; Coyne and Comstock, 2019; Garcia et al., 2016; Ghssein et al., 2016). Together, these known or proposed functions provide biological context for features observed in the Swarmium populations, including cell elongation, altered run-and-tumble behavior, and collective migration (Lee et al., 2017; Deochand and Grove, 2017), and identify potential mechanisms for future investigation.

One limitation of the current report is that—whereas genomic analysis identified mutations in several functional categories, including flagellar regulation, cell division, translation, and Type VI secretion-associated loci—these mutations were identified in genetically heterogeneous populations and their roles in functions pertinent to the swarming phenotype were not experimentally validated. Single-colony purification was not performed following serial passage because the objective of the planned study was to characterize the phenotypic and genomic changes arising in populations subjected to repeated swarming selection, rather than to isolate individual genotypes responsible for these changes. Future studies can build on these findings by isolating individual clones from the selected populations and determining whether the observed phenotypes and candidate variants are retained in defined clonal backgrounds. As such, it remains unclear whether these variants are drivers of the observed phenotype, compensatory adaptations, or secondary consequences of selection. We therefore emphasize that the genomic findings in this report are associative rather than causal. Subsequent targeted mutagenesis, complementation, and phenotypic characterization of defined clonal isolates will be necessary to establish the contributions of individual variants.

Several additional limitations should be considered. First, coding SNPs were not systematically classified as synonymous or non-synonymous, limiting mechanistic interpretation of the identified variants. Second, passage number differences between strains may introduce variability in background mutations. Third, the data presented in this report primarily reflect changes in individual cell motility parameters rather than quantifying swarming rate and direct measurements of collective swarming behavior. Whereas, the selection protocol utilized swarming conditions, bulk swarming dynamics were not quantitatively assessed. Thus, our findings are more accurately interpreted as describing motility changes in populations derived under swarming selection, rather than definitive alterations in swarming behavior itself. Finally, more rigorous analyses of motility (e.g., mean square displacement or angle autocorrelation) would strengthen conclusions regarding directional persistence.

In summary, this study establishes an experimental framework for linking laboratory selection, motility phenotypes, and genomic variation in *Enterobacter sp*. SM3. Our results demonstrate pronounced changes in bacterial morphology and motility in populations derived under repeated swarming selection and identify population-level genomic variation affecting several biologically relevant loci. Rather than identifying definitive mechanisms, these findings provide a foundation for future studies to establish genotype-phenotype relationships through defined clonal isolates and functional genetic approaches.

## Data Availability

The genomic data generated in this study have been deposited in the NCBI (https://www.ncbi.nlm.nih.gov) under BioProject accession PRJNA1530472. The BioSample accession numbers are SAMN63177463-SAMN63177474, corresponding to the parental SM3 sample and the five Swarmium populations (SM3SA-SM3SE). The microscopy and motility data supporting the conclusions of this study will be made available by the corresponding author upon request.

## References

[B1] Allison C. Coleman N. Jones P. L. Hughes C. (1992a). Ability of proteus mirabilis to invade human urothelial cells is coupled to motility and swarming differentiation. Infect. Immun. 60, 4740–4746. doi: 10.1128/iai.60.11.4740-4746.1992 1398984 PMC258226

[B2] Allison C. Lai H. C. Hughes C. (1992b). Co-ordinate expression of virulence genes during swarm-cell differentiation and population migration of *Proteus mirabilis*. Mol. Microbiol. 6, 1583–1591. doi: 10.1111/j.1365-2958.1992.tb00883.x 1495387

[B3] Ariel G. Rabani A. Benisty S. Partridge J. D. Harshey R. M. Be'er A. (2015). Swarming bacteria migrate by Lévy walk. Nat. Commun. 6:8396. doi: 10.1038/ncomms9396 26403719 PMC4598630

[B4] Ayan G. B. Park H. J. Gallie J. (2020). The birth of a bacterial tRNA gene by large-scale, tandem duplication events. eLife 9:e57947. doi: 10.7554/eLife.57947 PMC7661048 33124983

[B5] Berg H. C. (2003). The rotary motor of bacterial flagella. Annu. Rev. Biochem. 72, 19–54. doi: 10.1146/annurev.biochem.72.121801.161737 12500982

[B6] Berg H. C. Anderson R. A. (1973). Bacteria swim by rotating their flagellar filaments. Nature 245, 380–382. doi: 10.1038/245380a0 4593496

[B7] Bru J. L. Kasallis S. J. Zhuo Q. Høyland-Kroghsbo N. M. Siryaporn A. (2023). Swarming of *P. aeruginosa*: through the lens of biophysics. Biophys. Rev. 4:031305. doi: 10.1063/5.0128140 PMC10540860 37781002

[B8] Buddelmeijer N. Beckwith J. (2004). A complex of the *Escherichia coli* cell division proteins FtsL, FtsB and FtsQ forms independently of its localization to the septal region. Mol. Microbiol. 52, 1315–1327. doi: 10.1111/j.1365-2958.2004.04044.x 15165235

[B9] Copeland M. F. Weibel D. B. (2009). Bacterial swarming: a model system for studying dynamic self-assembly. Soft Matter 5:1174. doi: 10.1039/b812146j PMC3733279 23926448

[B10] Coyne M. J. Comstock L. E. (2019). Type VI secretion systems and the gut *microbiota. Microbiol*. Spectr. 7. doi: 10.1128/microbiolspec.PSIB-0009-2018 30825301 PMC6404974

[B11] Darnton N. C. Turner L. Rojevsky S. Berg H. C. (2010). Dynamics of bacterial swarming. Biophys. J. 98, 2082–2090. doi: 10.1016/j.bpj.2010.01.053 PMC2872219 20483315

[B12] De A. Chen W. Li H. Wright J. R. Lamendella R. Lukin D. J. . (2021). Bacterial swarmers enriched during intestinal stress ameliorate damage. Gastroenterology 161, 211–224. doi: 10.1053/j.gastro.2021.03.017 PMC8601393 33741315

[B13] Deochand D. K. Grove A. (2017). MarR family transcription factors: dynamic variations on a common scaffold. Crit. Rev. Biochem. Mol. Biol. 52, 595–613. doi: 10.1080/10409238.2017.1344612 28670937

[B14] Garcia E. C. Perault A. I. Marlatt S. A. Cotter P. A. (2016). Interbacterial signaling via Burkholderia contact-dependent growth inhibition system proteins. Proc. Nat. Acad. Sci. 113, 8296–8301. doi: 10.1073/pnas.1606323113 PMC4961174 27335458

[B15] Ghssein G. Brutesco C. Ouerdane L. Fojcik C. Izaute A. Wang S. . (2016). Biosynthesis of a broad-spectrum nicotianamine-like metallophore in Staphylococcus aureus. Science. 352, 1105–1109. doi: 10.1126/science.aaf1018 27230378

[B16] Grognot M. Taute K. M. (2021). More than propellers: how flagella shape bacterial motility behaviors. Curr. Opin. Microbiol. 61, 73–81. doi: 10.1016/j.mib.2021.02.005 33845324

[B17] Harvey K. L. Jarocki V. M. Charles I. G. Djordjevic S. P. (2019). The diverse functional roles of elongation factor Tu (EF-Tu) in microbial pathogenesis. Front. Microbiol. 10:2351. doi: 10.3389/fmicb.2019.02351 PMC6822514 31708880

[B18] Johnson S. Freedman B. Tang J. X. (2024). Run-and-tumble kinematics of *Enterobacter Sp*. SM3. Phys. Rev. E 109:064402. doi: 10.1103/PhysRevE.109.064402 39021001

[B19] Kamdar S. Ghosh D. Lee W. Tătulea-Codrean M. Kim Y. Ghosh S. . (2023). Multiflagellarity leads to the size-independent swimming speed of peritrichous bacteria. Proc. Nat. Acad. Sci. 120:e2310952120. doi: 10.1073/pnas.2310952120 PMC10691209 37991946

[B20] Kearns D. B. (2010). A field guide to bacterial swarming motility. Nat. Rev. Microbiol. 8, 634–644. doi: 10.1038/nrmicro2405 PMC3135019 20694026

[B21] Kearns D. B. Losick R. (2003). Swarming motility in undomesticated *Bacillus subtilis*. Mol. Microbiol. 49, 581–590. doi: 10.1046/j.1365-2958.2003.03584.x 12864845

[B22] Klappenbach J. A. Dunbar J. M. Schmidt T. M. (2000). rRNA operon copy number reflects ecological strategies of bacteria. Appl. Environ. Microbiol. 66, 1328–1333. doi: 10.1128/AEM.66.4.1328-1333.2000 PMC91988 10742207

[B23] Klappenbach J. A. Saxman P. R. Cole J. R. Schmidt T. M. (2001). rrndb: the ribosomal RNA operon copy number database. Nucleic Acids Res. 29, 181–184. doi: 10.1093/nar/29.1.181 PMC29826 11125085

[B24] Lee C. M. Monson R. E. Adams R. M. Salmond G. P. C. (2017). The LacI–family transcription factor, RbsR, is a pleiotropic regulator of motility, virulence, siderophore and antibiotic production, gas vesicle morphogenesis and flotation in serratia. Front. Microbiol. 8:1678. doi: 10.3389/fmicb.2017.01678 PMC5601083 28955306

[B25] Lee Y. Y. Belas R. (2015). Loss of FliL alters proteus mirabilis surface sensing and temperature-dependent swarming. J. Bacteriol. 197, 159–173. doi: 10.1128/JB.02235-14 PMC4288675 25331431

[B26] Majdalani N. Gottesman S. (2005). The Rcs phosphorelay: a complex signal transduction system. Annu. Rev. Microbiol. 59, 379–405. doi: 10.1146/annurev.micro.59.050405.101230 16153174

[B27] Mobley H. L. Belas R. (1995). Swarming and pathogenicity of proteus mirabilis in the urinary tract. Trends Microbiol. 3, 280–284. doi: 10.1016/S0966-842X(00)88945-3 7551643

[B28] Overhage J. Bains M. Brazas M. D. Hancock R. E. (2008). Swarming of Pseudomonas aeruginosa is a complex adaptation leading to increased production of virulence factors and antibiotic resistance. J. Bacteriol. 190, 2671–2679. doi: 10.1128/JB.01659-07 18245294 PMC2293252

[B29] Partridge J. D. Harshey R. M. (2013). Swarming: flexible roaming plans. J. Bacteriol. 195, 909–918. doi: 10.1128/JB.02063-12 23264580 PMC3571328

[B30] Partridge J. D. Nhu N. T. Q. N. Dufour Y. S. Harshey R. M. (2020). Tumble suppression is a conserved feature of swarming motility. mBio 11:e01189-20. doi: 10.1128/mBio.01189-20 32546625 PMC7298715

[B31] Partridge J. D. Nhu T. Q. N. Dufour Y. S. Harshey R. M. (2019). *Escherichia coli* remodels the chemotaxis pathway for swarming. MBio. 10:e00316-19. doi: 10.1128/mBio.00316-19 30890609 PMC6426603

[B32] Patrick J. E. Kearns D. B. (2012). Swarming motility and the control of master regulators of flagellar biosynthesis. Mol. Microbiol. 83, 14–23. doi: 10.1111/j.1365-2958.2011.07917.x PMC3245337 22092493

[B33] Pawul C. Dutta T. T. Johnson S. G. Tang J. X. (2024). Mucin promotes bacterial swarming by making the agar surface more slippery. Langmuir. 40, 27307–27313. doi: 10.1021/acs.langmuir.4c03395 39680870

[B34] Ping L. Wu Y. Hosu B. G. Tang J. X. Berg H. C. (2014). Osmotic pressure in a bacterial swarm. Biophys. J. 107, 871–878. doi: 10.1016/j.bpj.2014.05.052 25140422 PMC4142250

[B35] Sharma M. Anand S. (2002). Swarming: a coordinated bacterial activity. Curr. Sci. 83, 707–715.

[B36] Shneider M. M. Buth S. A. Ho B. T. Basler M. Mekalanos J. J. Leiman P. G. (2013). PAAR-repeat proteins sharpen and diversify the type VI secretion system spike. Nature 500, 350–353. doi: 10.1038/nature12453 PMC3792578 23925114

[B37] Turner L. Zhang R. Darnton N. C. Berg H. C. (2010). Visualization of flagella during bacterial swarming. J. Bacteriol. 192, 3259–3267. doi: 10.1128/JB.00083-10 20363932 PMC2897679

[B38] Verstraeten N. Debkumari K. B. Fauvart M. Fransaer J. Vermant J. Michiels J. (2008). Living on a surface: swarming and biofilm formation. Trends Microbiol. 16, 496–506. doi: 10.1016/j.tim.2008.07.004 18775660

[B39] Yang A. Tang W. S. Si T. Tang J. X. (2017). Influence of physical effects on the swarming motility of *Pseudomonas aeruginosa*. Biophys. J. 112, 1462–1471. doi: 10.1016/j.bpj.2017.02.019 PMC5389960 28402888

[B40] Zhang Z. Liu H. Karani H. Mallen J. Chen W. De A. . (2022). Enterobacter sp. strain SM1_HS2B manifests transient elongation and swimming motility in liquid medium. Microbiol. Spectr. 10:e02078–21. doi: 10.1128/spectrum.02078-21 35647691 PMC9241836

